# Experimental Analysis and Multiscale Modeling of the Dynamics of a Fiber-Optic Coil

**DOI:** 10.3390/s22020582

**Published:** 2022-01-13

**Authors:** Özkan Kahveci, Caner Gençoğlu, Tuncay Yalçinkaya

**Affiliations:** 1 Roketsan Missiles Industries Inc., Ankara 06780, Turkey; ozkan.kahveci@roketsan.com.tr (Ö.K.); cgencoglu@roketsan.com.tr (C.G.); 2 Department of Aerospace Engineering, Middle East Technical University, Ankara 06800, Turkey

**Keywords:** fiber, fiber-optic gyroscope, modal test, modal analysis, representative volume element, fiber-optic coil, finite element method, homogenization

## Abstract

Fiber-optic gyroscopes (FOGs) are common rotation measurement devices in aerospace applications. They have a wide range of diversity in length and in the winding radius of the coil to meet system requirements. Every dimensional parameter in the coil influences the dynamic response of the system, eventually leading to measurement errors. In order to eliminate the errors and to qualify the system, after the design and production stages, a deep and comprehensive testing procedure follows. In this study, the dynamic behavior of a quadrupole wound fiber-optic coil is investigated. First, pre-wound fiber-optic coils are tested with an impact modal test, where the mode shapes and natural frequencies are determined with structural data acquisition. For the modal analysis, a finite element (FE) model is developed where a representative volume element (RVE) analysis is also included to properly consider the influence of the microstructure. The experimental and numerical results are compared and validated. Moreover, an estimation model is proposed for a type of coil with different fiber lengths. Finally, the estimated coil set is produced and tested employing the same methodology in order to illustrate the capacity of the developed framework.

## 1. Introduction

Fiber-optic gyroscopes (FOGs) are rotation sensing devices utilizing Sagnac’s principle. They use phase difference between two counter traveling laser waves to compute the rotation motion of the body they are attached to [1,2,3]. FOG sensors work on a similar principle to ring laser gyroscopes (RLGs); however, FOGs offer many advantages over RLGs, such as low cost, low weight, low power consumption, no precise machining requirement, no gas medium or leakage problems, and no vibratory mechanism to overcome lock-in problems at low rotation rates [4,5]. In recent decades, FOG technology has developed rapidly owing to advances in low-loss fiber-optic cables, light sources, and detectors used in the telecommunications industry [1,4,6]. A FOG sensor is mainly composed of a light source, a coupler, a phase modulator, a photo detector, and, most essentially, a coil of fiber-optic cable [3,7,8,9].

In order to ensure the precision of a FOG sensor, maintaining the symmetrical loading for the clockwise and counterclockwise paths along which light travels is crucial for the performance. Due to the effects of thermal and mechanical stresses on a FOG coil, the refractive index of light differs in the fiber-optic cable causing an unsymmetrical loading difference between the clockwise and counterclockwise paths that light travels along, eventually resulting in a bias error at the sensor readout [1,10]. In the event of a time-dependent temperature gradient along the fiber, non-reciprocity bias errors known as the Shupe effect arise [11]. As a solution to this problem, symmetric coil-winding methods are suggested [12]. A commonly preferred method is the “quadrupole” winding pattern, yet other novel patterns and methods to overcome non-reciprocity errors are being studied in the literature [13,14,15]. The errors due to temperature effects in a FOG sensor are able to be analytically modeled, and these errors can be compensated from the sensor output by employing proper algorithms [16,17,18,19,20]. The utilization of the finite element (FE) method is an alternative approach to the analytical modeling of disturbance effects due to temperature variations on FOG coils [21,22,23,24,25].

Mechanical loadings are as important as thermal loadings on the performance of FOG sensors. The success of a FOG sensor is related to low stress levels on the fiber-optic cable wound on the coil. There are studies in the literature that focus on addressing methods for reducing stress on FOG coils during the winding process [8,26,27]. Other than winding processes, one source of stress in FOG coils is mechanical vibrations. Modeling and compensating the effects of mechanical vibrations has been the subject of certain studies [28,29,30]. In order to reduce the vibration-based errors and identify the characteristics of the system, the FE theory has also been used to determine the resonance frequency of the structure to enhance the performance of FOGs [31,32]. FE modeling of the mechanics of rotations sensors is also utilized for RLG sensors as well as FOG sensors [33]. Thanks to FE methodology, deflections and stress distributions on the sensitive parts of optical sensors working with the Sagnac principle can be computed accurately [34,35].

In the finite element analysis (FEA) of FOG coil conducted here, a meaningful section of the coil, including a single fiber with a fiber core, cladding, glue, and sections of neighboring cables, is modeled in an appropriate symmetry boundary condition to represent the global behavior of the coil material. This section is called the representative volume element (RVE) and a homogenization procedure is followed where the coil material properties are obtained from the RVE [20,22,23,25,36,37]. FOG coil is composed of a fiber-optic cable in a staggered formation glued to each other by adhesives in an orderly fashion. The fiber-optic cable is composed of a fiber core in which light travels and the fiber core is covered by protective cladding [7,8,19,22,38,39]. Since the coil structure is composite, the methods of homogenization for modeling the mechanical and thermal properties of composite structures also hold for the FOG coil, and in this study, such an approach is followed [40,41,42,43,44,45,46,47].

The studies that include an FEA of FOG coils mostly tackle thermal loading problems; however, a study in which the mechanical modeling of a FOG coil with the FE and RVE methods and the results verified using vibration testing is rare. In the literature, the computation of the orthotropic elastic properties of composite structures utilizing impact hammer modal testing and FE analysis has been addressed [48]. In this study, the mechanical characterization of a FOG coil has been extracted by employing the RVE approach using FEA. With proper material properties, the RVE model of the FOG coil is established. Through the RVE model, obtained homogenized orthotropic elastic properties are used in a solid FOG coil FE model to calculate the natural frequencies and mode shapes of the coil. These vibrational properties of the FOG coil from the FEA are verified by the data obtained through impact hammer modal testing. For the modal testing, the coil structure is suspended using soft elastic rubbers to make free boundary conditions, the smallest possible accelerometers have been used to reduce the mass coupling effect, and the excitation is generated by an impact hammer attached to the automated modal test robot (MTR) in order not to make the coupling between the FOG coil and the exciter [49,50]. Moreover, an estimation model is proposed and tested against other coils with different dimensions. A different coil type is produced and tested, and the estimation of the FE model is verified by testing the new coil batch. It is important to point out that the verification of the homogenized material properties of FOG coils by means of impact modal testing is a novelty for the literature. In order to accomplish this, a scientific approach has been developed for a practical engineering problem. Thanks to this method, researchers have the opportunity to assess the dynamic behavior of a newly designed FOG coil prior to cumbersome testing processes. This complete multiscale modeling with experimental verification is a unique study for the literature.

FOG coil has been modeled to be perfectly symmetric, and with such structures, double modes are expected at the same frequency as two orthogonal mode shapes [50]. Due to the nature of the quadrupole winding method, the jogging zone causes these twin modes to occur slightly separated, with the nodal lines of these modes being fixed. Using modal testing and a finite element analysis approach, a FOG model with verified orthotropic elastic properties is obtained.

There is a fiber optic coil which will be called Coil Type-1 in this study. Based on the existing gyro parameters of Coil Type-1, a research study started to develop a different grade product with a required total fiber length of 450 m, which will be named in this document as Coil Type-2 and have approximately half of the total fiber length of Coil Type-1. At this stage, the vibrational characteristics of the developing fiber-optic gyroscope need to be studied. The workflow of the study begins with creating the RVE model for the composite coil structure, which consists of the fiber, the coating layer of the fiber, and the adhesive applied between the fibers. The material properties are taken from the supplier datasets and the RVE parameters are then calculated in the sequence, as explained in Section 3. After the modal tests for the Type-1 coil batch, the global FE model is created and the results are compared with the test results. The RVE parameters and the global cylindrical FE model are set, and from the global model of the Type-1 coil, the global model for the Coil Type-2 is established and the dynamic properties of the Type-2 coils are estimated. Later, the Type-2 coil batch is produced and the same modal test procedure is applied. The cylindrical global FE model for the coil structure uses the verified RVE parameters as input and the geometric features as parameterized properties to give the designer the freedom to decide on further design configurations based on the changing fiber length using the dynamic response of the coil. By fitting the FE model, the designer can decide on the geometric properties and materials that the coil support configuration should have for new coil structures.

The paper is organized as follows. First, in Section 2, the material properties of fiber, adhesive, and coating are presented. In addition, the modal testing procedure is explained for each coil configuration. Next, in Section 3, the computational framework of the RVE parameters are given for a unit cell. The modal test results for both coil configurations are discussed in Section 4. The global FE analysis results are discussed in Section 5. Finally, the conclusion of the work is presented in Section 6.

## 2. Materials and Modal Test Methodology

The modal testing approach is utilized to analyze the composite coil structure for Coil Type-1 and Coil Type-2. The results of these modal tests will be discussed in Section 4 for both coil configurations.

Regarding fiber material, the technical specifications are given in Table 1. In addition, Epotek^®^ 330 is chosen as the coil adhesive due to its extremely wide application in gyroscopes and in aerospace industries. The coils are produced with the quadrupole winding method. The dimensions of the core, cladding, and coating parts are 6.5, 80, and 155 μm, respectively. 

**Figure 1:**
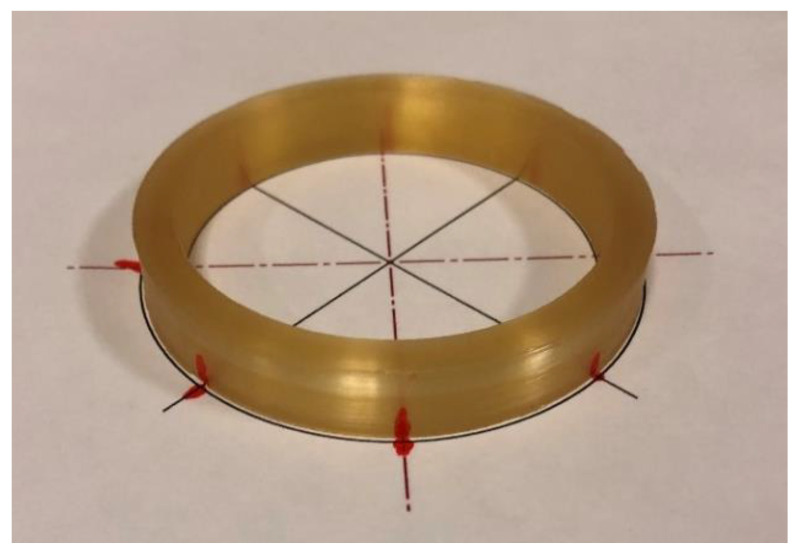
Type-1 fiber-optic coil.

The geometric parameters of Coil Type-1can be seen in Table 2. There are five identical coils with the same dimensions for the tests, and the results are the average values of the test batch (Figure 1).

The aim of this experimental study is to collect data on the FOG coil in order to analyze the dynamic behavior of the coil and mode shapes of the composite coil structure. In impact hammer modal testing, the impact hammer and accelerometer time domain measurements are acquired simultaneously upon impact. The hammer provides an impact to the structure to excite the modes as an input, and a necessary and sufficient number of accelerometers collect the vibration acceleration data from the structure as an output. A frequency response function (FRF) is obtained by dividing the Fourier transform of the accelerometer data by the hammer data with respect to frequency. The FRF serves as a transfer function between excitation and response. With the help of a measured set of FRFs, one can obtain the natural frequencies, mode shapes, and damping ratios of a physical structure for a selected set of modes by a procedure called experimental modal analysis (EMA) [49,50]. In order to observe the mode shapes of the structure, a sufficient amount of reference points must be used for data acquisition. From FRF plots, the resonant frequencies of a structure can be spotted and the damping ratios can be deduced. The damping of the structure at the peaks in the FRF graph is proportional to the width of the peaks. The wider the peak, the heavier the damping. Tests are performed using the LMS SCADAS data acquisition system. Before the modal tests, eight locations are marked on the coils with 45-degree angles around the radial direction. Red dots show the hammer-hitting locations and the accelerometer positions (Figure 1).

**Figure 2:**
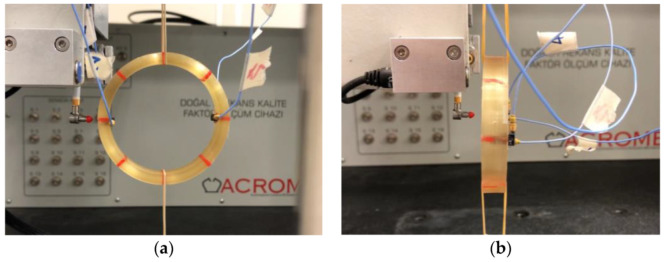
The free-free boundary condition for the modal test.

Tests are carried out in two stages, representing the hitting directions of the hammer to excite the structure in different directions to better uncover the modal shapes. In Figure 2, both hitting directions can be seen when the impact hammer of the modal test robot is located in the hoop and the axial direction. In the modal tests, four one-axis accelerometers are placed on two axial and two rear (hoop) sides of the coil. Points are numbered from 1 to 8 on the coil substances by 45-degree intervals along the circumference of the coil. Point number 1 is the hammer location in the tests.

At the first stage, the impact hammer is placed on the rear (hoop) side of point 1, while the 4 accelerometers are located at points 1 and 5 at the hoop and axial directions (Figure 2a). The accelerometers are then moved to points 2 and 6, points 3 and 7, and points 4 and 8, respectively. Next, the first stage of the modal test is completed by the fixed hammer location at the hoop side excitation.

At the second stage, the impact hammer excitation is generated from the axial side of the coils (Figure 2b). When the hammer is placed at point 1, the accelerometers are placed at points 1 and 5 at the hoop and axial axis directions, and the moving accelerometer procedure follows as per the first stage.

The impact hammer triggers the mode shape of the structure in the same direction where it is excited, while the mode shapes of the other direction are not triggered enough, so the excitation must be given in two directions. Merging the data from each excitation test produces the best behavior for the coil samples if they are evaluated in the same basket. The mode shapes of these structures are displayed with this method.

Type-2 coils are produced with the same quadrupole winding method. The fibers used in the Type-2 coil batch are the same with Type-1 coil batch. The geometric parameters of Type-2 coils can be seen in Table 3. There are five identical coils with the same dimensions for the tests, and the results are the average values of the test batch. The same two-stage testing process is also carried out for the Type-2 coil batch.

**Figure 3:**
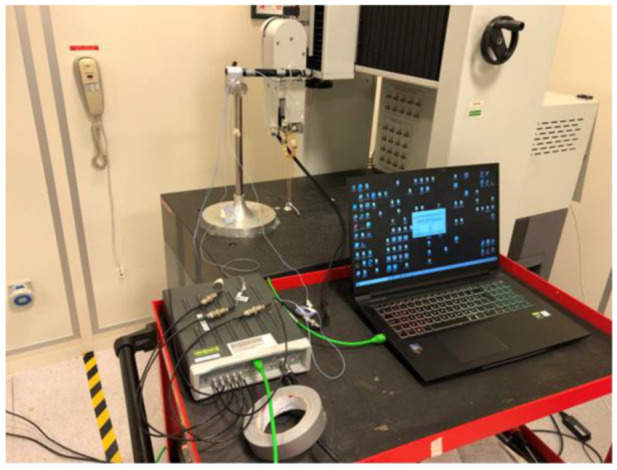
Modal test robot and data acquisition test setup.

The tests are carried out with ICP-type single-axis mini PCB-Piezotronics accelerometers for all configurations. There are eight accelerometers available that have average actual sensitivities of approximately 5.2 mV/g. The data acquisition system has eight channel inputs, and one channel is reserved for the impact hammer, so there are seven channels left to the accelerometers. Therefore, one impact hammer and four accelerometers are used to carry out the tests. The structures acquisition module of LMS Test Lab software is used to process the acquired data (Figure 3).

Fiber-optic coils are produced with the automated winding machine on a mandrel with two housing Teflon disks at both ends through the manual guidance of the technician to the fibers. Throughout the winding process, the adhesive between the fibers is applied with a brush, so the coils have small structural differences, causing slight dynamic behavioral differences for each coil. In order to minimize data collection errors, an automated modal test robot is used during the data acquisition process, and the excitation given to the structure is kept stable.

## 3. Finite Element Modeling

In this study, FEA is employed with an RVE approach in order to obtain the equivalent material properties of a fiber-optic coil wound by a quadrupole winding method. In order to establish the mechanical model, the material behavior needs to be identified. The classical representation of linear orthotropic elasticity states that 21 independent constants of the 6 × 6 stiffness matrix, as shown in Equation (1), need to be reduced from the most general anisotropic material to the real case:

(1) $$\left\{\begin{array}{c} \begin{array}{c} R_{1}\\ R_{2}\\ R_{3} \end{array}\\ \begin{array}{c} R_{4}\\ R_{5}\\ R_{6} \end{array} \end{array}\right\}=\left[\begin{array}{cc} \begin{array}{ccc} \begin{array}{c} \begin{array}{c} K_{11}\\ K_{21}\\ K_{31} \end{array}\\ \begin{array}{c} K_{41}\\ K_{51}\\ K_{61} \end{array} \end{array} & \begin{array}{c} \begin{array}{c} K_{12}\\ K_{22}\\ K_{32} \end{array}\\ \begin{array}{c} K_{42}\\ K_{52}\\ K_{62} \end{array} \end{array} & \begin{array}{c} \begin{array}{c} K_{13}\\ K_{23}\\ K_{33} \end{array}\\ \begin{array}{c} K_{43}\\ K_{53}\\ K_{63} \end{array} \end{array} \end{array} & \begin{array}{ccc} \begin{array}{c} \begin{array}{c} K_{14}\\ K_{24}\\ K_{34} \end{array}\\ \begin{array}{c} K_{44}\\ K_{54}\\ K_{64} \end{array} \end{array} & \begin{array}{c} \begin{array}{c} K_{15}\\ K_{25}\\ K_{35} \end{array}\\ \begin{array}{c} K_{45}\\ K_{55}\\ K_{65} \end{array} \end{array} & \begin{array}{c} \begin{array}{c} K_{16}\\ K_{26}\\ K_{36} \end{array}\\ \begin{array}{c} K_{46}\\ K_{56}\\ C_{K_{66}} \end{array} \end{array} \end{array} \end{array}\right]=\left\{\begin{array}{c} \begin{array}{c} D_{1}\\ D_{2}\\ D_{3} \end{array}\\ \begin{array}{c} D_{4}\\ D_{5}\\ D_{6} \end{array} \end{array}\right\}$$

The most general f=K∗u matrix equation will be reduced to a particular class of anisotropy, i.e., orthotropy by using the invariance equations. The reduction of this matrix depends on the mutually orthogonal planes and the symmetry group of the condition. The equations are simplified in the matrix-vector notation. The solution requires Voigt tensor notation together with the invariance conditions. If there is a unidirectional composite with circular fibers arranged in a rectangular array, the elasticity tensor will not be aligned with the shear direction and the zero components of the stiffness matrix will not be zero anymore. Therefore, the normal stresses will induce shear strains. The unidirectional composite with circular fibers in a square array is the best example of this situation. In these materials, there is a single material direction, and in the planes that are orthogonal to this direction, the material is isotropic. In this kind of transversely isotropic linear elasticity case, the elasticity tensor has five independent coefficients (Equation (2)). The symmetry group contains all rotations around a given material direction:

(2) $$\left[\begin{array}{cc} \begin{array}{ccc} \begin{array}{c} \begin{array}{c} C_{11}\\ C_{12}\\ C_{12} \end{array}\\ \begin{array}{c} 0\\ 0\\ 0 \end{array} \end{array} & \begin{array}{c} \begin{array}{c} C_{12}\\ C_{11}\\ C_{12} \end{array}\\ \begin{array}{c} 0\\ 0\\ 0 \end{array} \end{array} & \begin{array}{c} \begin{array}{c} C_{12}\\ C_{12}\\ C_{11} \end{array}\\ \begin{array}{c} 0\\ 0\\ 0 \end{array} \end{array} \end{array} & \begin{array}{ccc} \begin{array}{c} \begin{array}{c} 0\\ 0\\ 0 \end{array}\\ \begin{array}{c} C_{44}\\ 0\\ 0 \end{array} \end{array} & \begin{array}{c} \begin{array}{c} 0\\ 0\\ 0 \end{array}\\ \begin{array}{c} 0\\ C_{44}\\ 0 \end{array} \end{array} & \begin{array}{c} \begin{array}{c} 0\\ 0\\ 0 \end{array}\\ \begin{array}{c} 0\\ 0\\ C_{44} \end{array} \end{array} \end{array} \end{array}\right]$$

**Figure 4:**
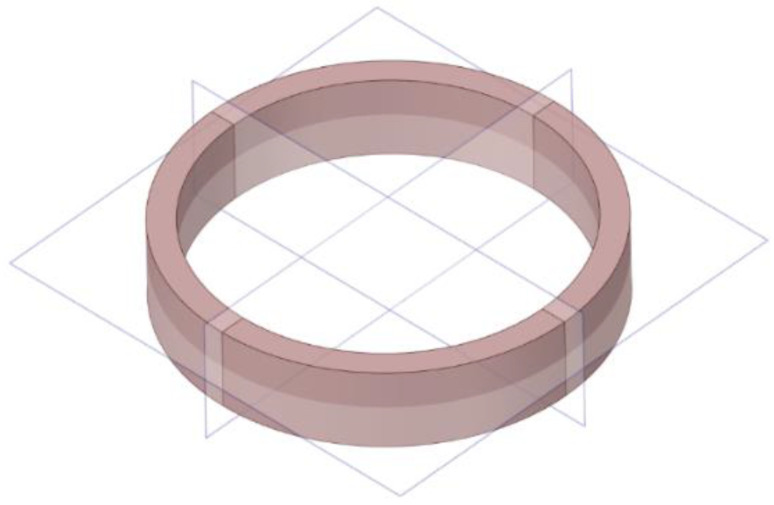
Symmetry planes of the fiber-optic coil.

It is known that the FOG coil is more flexible in the axial and radial directions where the adhesive matrix which stick the fibers together is more resistant in the hoop direction. The material properties of the FOG coil have three symmetry planes, as shown in Figure 4. Therefore, the FOG coil exhibits cubic orthotropic properties.

In this study, an FE model is established using ABAQUS CAE software. The coil is taken as a composite structure. The model uses a homogenization method which is based on modeling the microstructure, and applies the technique to obtain the properties of the composite with the micromechanics method. In the FE model, a representative volume element (RVE) is created as a microstructure, and the global parameters of the composite coil structure are found by this local RVE analysis.

Firstly, the RVE model of the composite structure is a cross-sectional cut of the FOG coil and is considered to have cylindrical fibers of infinite length, which are filled with the adhesive. The cross-section of the structure is obtained by cutting with an orthogonal plane to the fiber axis. This representative section has a periodic microstructure, so it can be used in FE analysis as a three-dimensional RVE. Normally, parallel fibers with an adhesive reinforced structure show orthotropic material properties at the lamina level. In the cases where hexagonal arrays occur, the properties become transversely isotropic [41].

**Figure 5:**
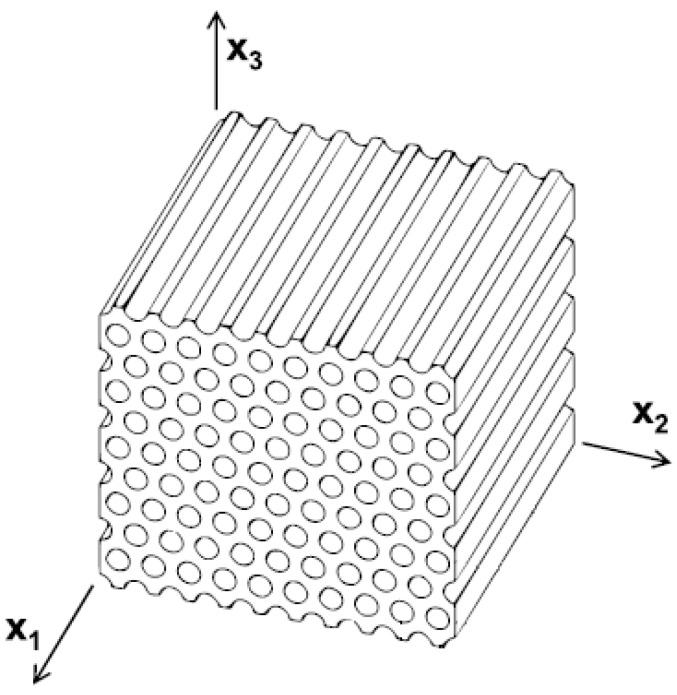
Cross-section of the composite material sample [41].

The sample shown in Figure 5 is an example cross-sectional model for the composite structure. This structure is similar to the coil fiber arrangement in real gyro applications.

**Figure 6:**
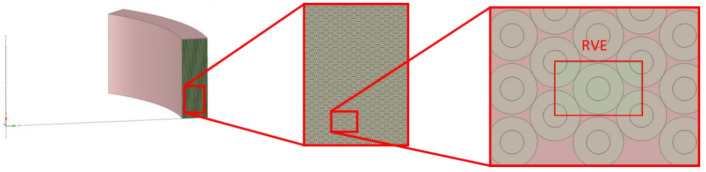
Selected representative volume element (RVE) cell from the global coil body.

In Figure 6, the selected RVE can be seen from the global coil structure of the FE analysis.

**Figure 7:**
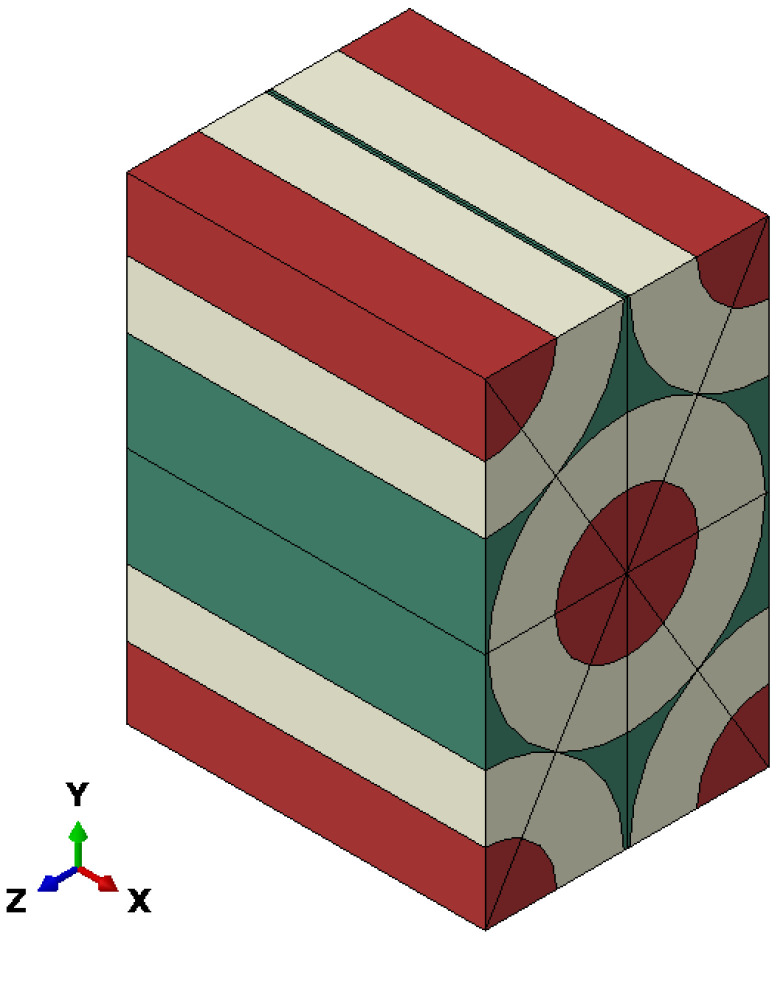
Representative volume element (RVE) selected for the actual coil case.

The RVE selected in this study is shown in Figure 7. The red color represents the glass sections, the beige color illustrates the coating of the fibers, and the green color shows the adhesive. The dimensions for the RVE are 266, 158, and 200 μm as the width (Y), height (Z), and depth (X), respectively, for the fiber introduced in Table 1.

The body equation and the stiffness matrix for the structure shown in Figure 7 is given in Equation (3) as:

(3) $$\left\{\begin{array}{c} \begin{array}{c} {\bar{\sigma }}_{1}\\ {\bar{\sigma }}_{2}\\ {\bar{\sigma }}_{3} \end{array}\\ \begin{array}{c} {\bar{\sigma }}_{4}\\ {\bar{\sigma }}_{5}\\ {\bar{\sigma }}_{6} \end{array} \end{array}\right\}=\left[\begin{array}{cc} \begin{array}{ccc} \begin{array}{c} \begin{array}{c} C_{11}\\ C_{12}\\ C_{12} \end{array}\\ \begin{array}{c} 0\\ 0\\ 0 \end{array} \end{array} & \begin{array}{c} \begin{array}{c} C_{12}\\ C_{22}\\ C_{23} \end{array}\\ \begin{array}{c} 0\\ 0\\ 0 \end{array} \end{array} & \begin{array}{c} \begin{array}{c} C_{12}\\ C_{23}\\ C_{22} \end{array}\\ \begin{array}{c} 0\\ 0\\ 0 \end{array} \end{array} \end{array} & \begin{array}{ccc} \begin{array}{c} \begin{array}{c} 0\\ 0\\ 0 \end{array}\\ \begin{array}{c} \left(C_{22}-C_{23}\right)/2\\ 0\\ 0 \end{array} \end{array} & \begin{array}{c} \begin{array}{c} 0\\ 0\\ 0 \end{array}\\ \begin{array}{c} 0\\ C_{66}\\ 0 \end{array} \end{array} & \begin{array}{c} \begin{array}{c} 0\\ 0\\ 0 \end{array}\\ \begin{array}{c} 0\\ 0\\ C_{66} \end{array} \end{array} \end{array} \end{array}\right]\left\{\begin{array}{c} \begin{array}{c} {\bar{\epsilon }}_{1}\\ {\bar{\epsilon }}_{2}\\ {\bar{\epsilon }}_{3} \end{array}\\ \begin{array}{c} {\bar{\gamma }}_{4}\\ {\bar{\gamma }}_{5}\\ {\bar{\gamma }}_{6} \end{array} \end{array}\right\}$$

After finding the components of transversely isotropic tensor C, the longitudinal and transverse Young’s moduli $E_{1}$ and $E_{2}$, the longitudinal and transverse $\nu_{12}$ and $\nu_{13},$ and the longitudinal shear modulus $G_{12}$ can be computed. In order to obtain the five elastic properties of the homogenized material given in Equation (3), the following equations can be used:

(4) $$E_{1}=C_{11}-2C_{12}^{2}/\left(C_{22}+C_{23}\right)$$

(5) $$\nu_{12}=C_{12}/\left(C_{22}+C_{23}\right)$$

(6) $$E_{2}=\left[C_{11}\left(C_{22}+C_{23}\right)-2C_{12}^{2}\right]\left(C_{22}-C_{23}\right)/\left(C_{11}-C_{12}^{2}\right)$$

(7) $$\nu_{23}=\left[C_{11}C_{23}-C_{12}^{2}\right]/\left(C_{11}C_{22}-C_{12}^{2}\right)$$

(8) $$G_{12}=C_{66}$$

In addition, the shear modulus $G_{23}$ can be calculated by:

(9) $$G_{23}=C_{44}=\frac{1}{2}\left(C_{22}-C_{23}\right)$$

Furthermore, the RVE should be subjected to displacement boundary conditions in order to obtain the overall elastic matrix *C* in every direction. The determination of the elements for the *C* tensor, the components of $C_{ij}$, can be found one by one throughout the columns. The boundary conditions to find the elements of first column of tensor *C* shows the displacement boundary condition in the fiber direction as:

(10) $$\epsilon_{1}^{0}=1\;\mathrm{and}\;\epsilon_{2}^{0}=\epsilon_{3}^{0}=\gamma_{4}^{0}=\gamma_{5}^{0}=\gamma_{6}^{0}=0$$

(11) $$\epsilon_{2}^{0}=1\;\mathrm{and}\;\epsilon_{1}^{0}=\epsilon_{3}^{0}=\gamma_{4}^{0}=\gamma_{5}^{0}=\gamma_{6}^{0}=0$$

The third column can be calculated from the first and second columns due to the transverse isotropy, but the components $C_{\alpha 3}$ for α = 1, 2, and 3 can be calculated by applying:

(12) $$\epsilon_{3}^{0}=1\;\mathrm{and}\;\epsilon_{1}^{0}=\epsilon_{2}^{0}=\gamma_{4}^{0}=\gamma_{5}^{0}=\gamma_{6}^{0}=0$$

In the first three columns, a unity displacement boundary condition is applied in positive X, Y, and Z directions, respectively. The rest of the surfaces are subjected to symmetry boundary conditions.

In the fourth column, only the term $C_{44}$ is different from zero and can be calculated as mentioned above in Equation (9). Different from the first three columns, there is a symmetry boundary condition in the Z direction and a periodic boundary condition in the X direction. The displacement is applied from the +Y surface of the RVE cell in the direction of +X. In the fifth column, only the term $C_{55}$ is different from zero and is equal to term $\;C_{66}$. In the sixth column (and in the fourth and fifth columns), the applied load is not symmetric, so the boundary conditions must be coupling type constraints, called tie constraints in the ABAQUS software. Thus, there is a symmetry boundary condition in the Y direction and a periodic boundary condition in the X direction. The displacement is applied from the +Z surface of the cell in the direction of +X.

**Figure 8:**
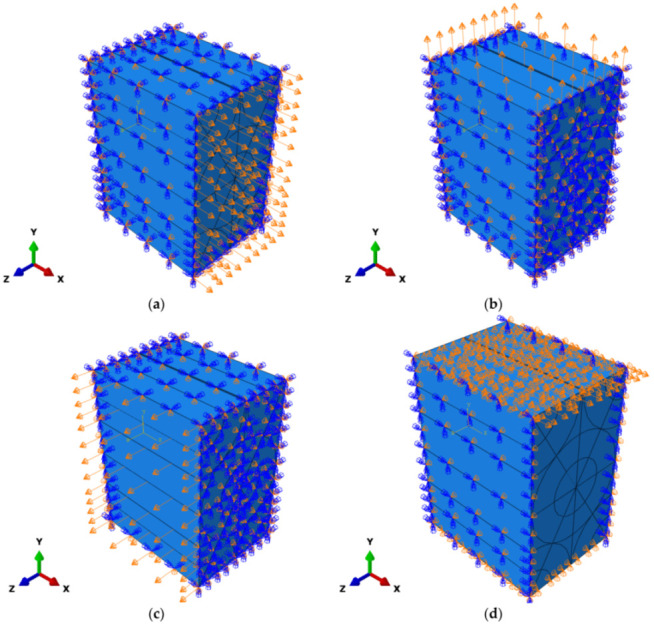

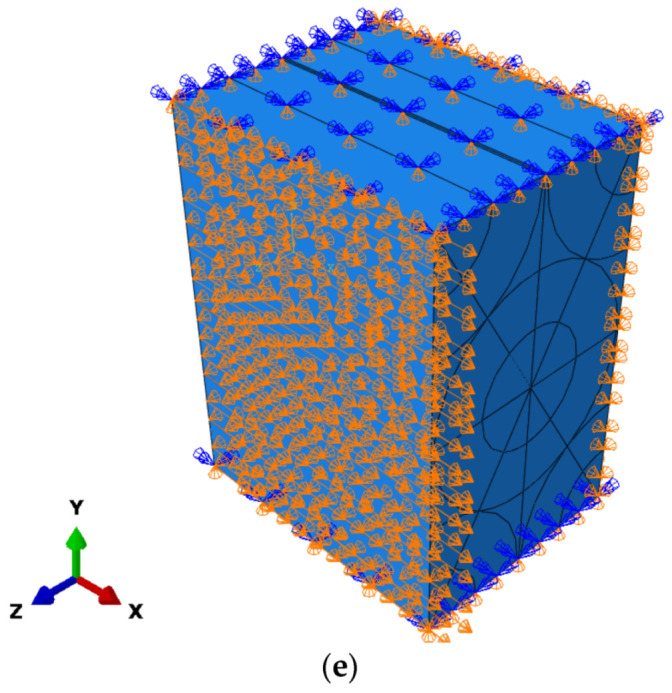
The boundary condition for the solution of the: (**a**) first column of tensor C; (**b**) second column of tensor C; (**c**) third column of tensor C; (**d**) fourth column of tensor C; and (**e**) sixth column of tensor C.

It can be seen from Figure 8a–c that applied boundary conditions for the calculation of the first three columns are also unity displacements of the RVE cell, according to Equation (3). For the fourth column, a periodic boundary condition is applied in the X direction, while there is a symmetry boundary condition applied along the Z direction. The displacement is applied in the direction of +X from the +Y surface of the RVE cell, as shown in Figure 8d, to calculate the shear modulus value defined in Equation (9). The fifth and sixth columns shown in Figure 8e are equal to each other, and they need a “tie constraint” boundary condition similar to the fourth column. In order to calculate the terms in the fifth and sixth columns, the displacement is applied from the +Z surface of the cell in the direction of +X. There is a symmetry boundary condition in the Y direction and a periodic boundary condition in the X direction.

A standard linear element type is used in the RVE cell model. In total, 24750 C3D8R elements are used in the RVE. The C3D8R element has eight node linear bricks and reduced integration with hourglass control. The degree of freedom of this element is 24. This element can be used in static deflection analysis [51].

## 4. Evaluation of Dynamic Test Results

In this section, the results of the modal tests for the Type-1 and the Type-2 coils will be discussed, respectively.

### 4.1. Modal Test Results of Coil Type-1

The mode shapes and resonance frequencies for the Type-1 coil will be evaluated. In the testing scheme, the axis definitions for a coil sample are: +X is the outward radial direction; and –X is the direction towards the center of the coil circle. +Z and –Z give the direction out-of-plane when the coil is placed on the table, as shown in Figure 1.

**Figure 9:**
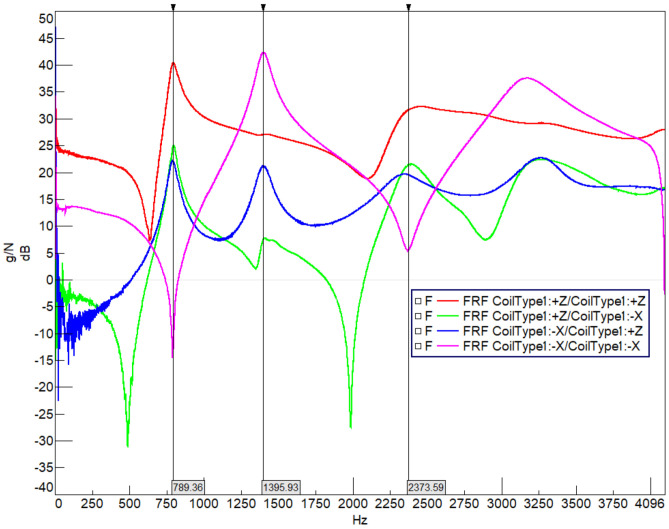
Drive point frequency response function (FRF) for Coil Type-1.

Figure 9 shows the drive point FRF result graph for a Type-1 coil. The drive point measurement is when the given excitation and collected data are at the same reference point and direction. The OX axis is frequency (Hz) and the OY axis is frequency response (g/N) shown in decibels (dB). The input from the hammer at point 1 and the response output from the accelerometer at point 1 in the –X direction (pink function) gives the modes in the radial direction mode shape frequencies as the peaks. This means the pink function shows an in-plane mode shape and a resonance movement at 1395 Hz, while the 789 Hz and 2373 Hz show an anti-resonance. Therefore, it can be seen from the FRF that there will be a radial movement of the coil structure as the second mode, while the first and third modes are oriented in an out-of-plane (Z) direction. In addition, the red line is the FRF curve for the out-of-plane (Z) excitation and response. The main peak points for the structure occur at the 789 Hz and 2373 Hz values as the first and third modes, so the out-of-plane bending modes are located at these frequencies.

**Figure 10:**
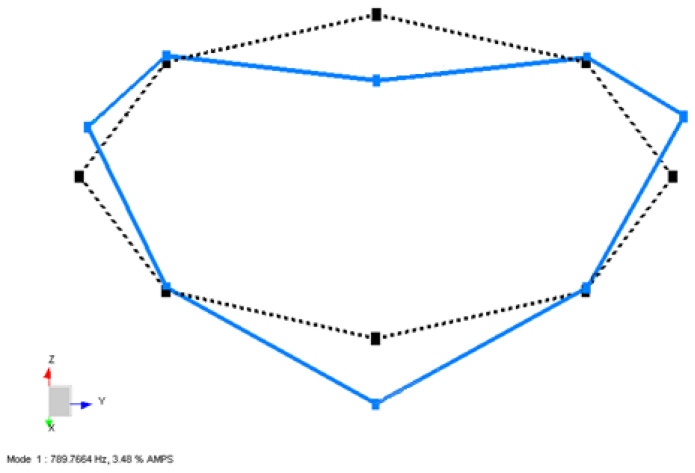
First mode shape obtained from LMS Test Lab software for a Type-1 coil sample.

**Figure 11:**
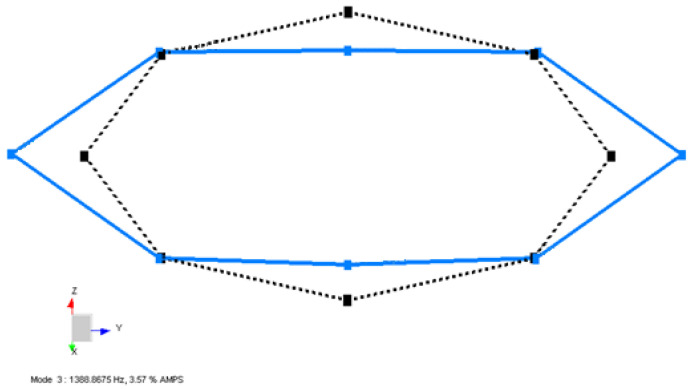
Second mode shape obtained from LMS Test Lab software for a Type-1 coil sample.

**Figure 12:**
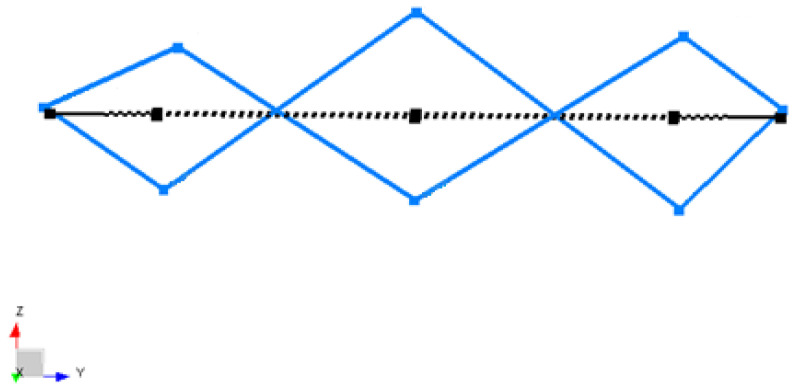
Third mode shape obtained from LMS Test Lab software for a Type-1 coil sample.

The blue lines represent the deformed shape, while the black dashed lines represent the undeformed shape of the Coil Type-1 in Figure 10, Figure 11 and Figure 12.

In Figure 10, the first bending mode of coil structure is obtained by processing both the –X and –Z excitation data. The excitation test results are merged into one complete data set to calculate the global natural frequencies and mode shapes. As expected, the first mode shape is a bending mode occurring at 789.7 Hz, as shown in Figure 10. The mode shape of the structure occurred bending from two points. These modes occur as twins close to each other, but not the exact same value, due to the transition zone of the quadrupole winding method, which causes the real case not to be perfectly symmetric.

In Figure 11, the first in-plane mode of coil structure is obtained by processing both the –X and –Z excitation data. The excitation test results are merged into one complete data set to calculate the global natural frequencies and mode shapes. As expected, the second mode shape occurred in the XY plane and it is placed at 1388.9 Hz, as shown. These modes occur as twins close to each other, but not the exact same value, due to the transition zone of the quadrupole winding method, which causes the real case not to be perfectly symmetric.

In Figure 12, the second out-of-plane mode of coil structure is obtained by processing both the –X and –Z excitation data. The excitation test results are merged into one complete data set to calculate the global natural frequencies and mode shapes. The third mode shape occurred as the second bending mode at 2375 Hz, as shown. The shape of the structure bends from three points. These modes occur as twins close to each other due to the transition zone of the quadrupole winding method. However, it is very difficult to monitor the three-point out-of-plane bending with four moving accelerometers placed at 45-degree intervals. Luckily, in this test, the merged excitation data provides a result closer to the real situation.

The mode shapes and resonance frequencies found for the Type-1 coil batch from the modal tests will be compared with the RVE-based global FE model for the Type-1 coil configuration. If they are compatible, the geometry of the FE model will be set to a Type-2 coil configuration and will be tested with the same procedure as stated in Section 2 to verify the RVE-based FE model by taking the material properties and the previously calculated fixed RVE parameters.

### 4.2. Modal Test Results of Coil Type-2

The mode shapes and resonance frequencies for the Type-2 coil will be discussed, and these results will be compared with the RVE-based global FE model for a Type-2 configuration in Section 5.2 to verify the global FE model based on the RVE method.

In the testing scheme, the axis definitions for the coil sample are: +X is the outward radial direction; and –X is the direction towards the center of the coil circle. +Z and –Z give the out-of-plane direction when the coil is placed on the table, as shown in Figure 1.

**Figure 13:**
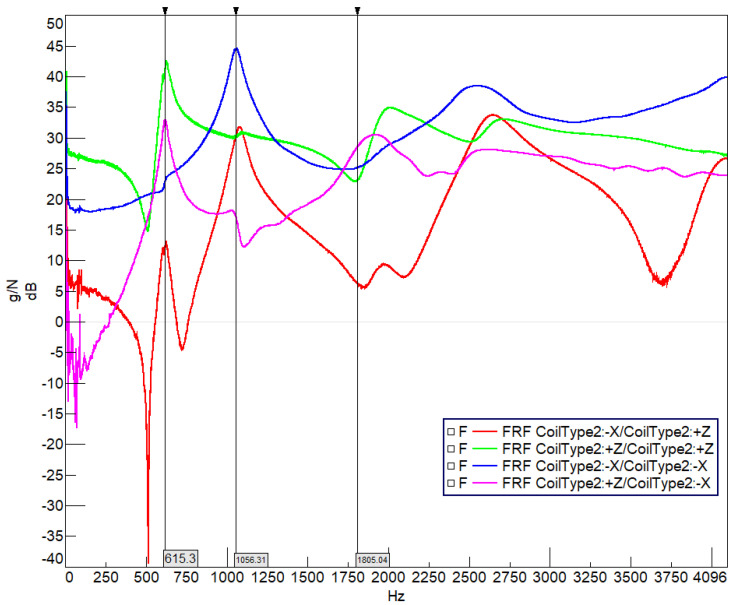
Drive point FRF for Coil Type-2.

Figure 13 illustrates the drive point FRF result graph for a Type-2 coil. The OX axis is frequency (Hz) and the OY axis is frequency response (g/N) shown in decibels (dB). The input from the hammer at point 1 and the response output from the accelerometer at point 1 in the –X direction (blue function) provides the modes in the radial direction mode shape frequencies as the peaks. This means the pink function shows an in-plane mode shape and a resonance movement at 1056 Hz, while the 615 Hz and 1805 Hz values show an anti-resonance. Therefore, it can be seen from the FRF that there will be a radial movement of the coil structure as the second mode, while the first and third modes are oriented in an out-of-plane (Z) direction. In addition, the green line is the FRF curve for the out-of-plane (Z) excitation and response. The main peak point for the structure occurs at the 615 Hz and 1805 Hz values as the first and third modes, so the out-of-plane bending modes are located at these frequencies.

**Figure 14:**
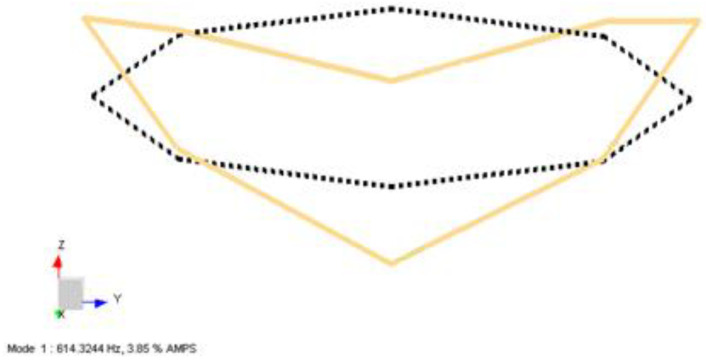
First mode shape obtained from LMS Test Lab software for the Type-2 coil sample.

**Figure 15:**
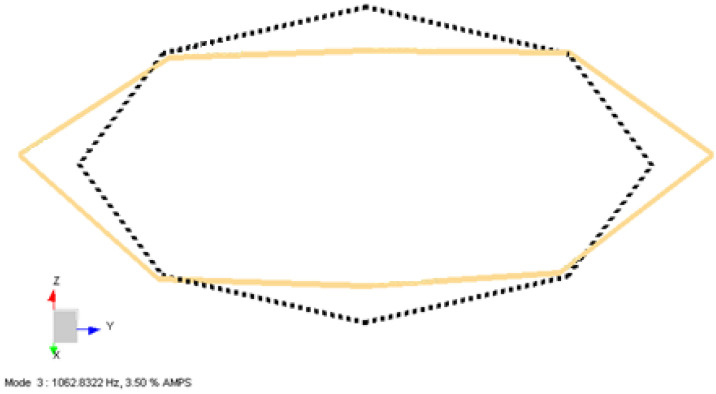
Second mode shape obtained from LMS Test Lab software for the Type-2 coil sample.

**Figure 16:**
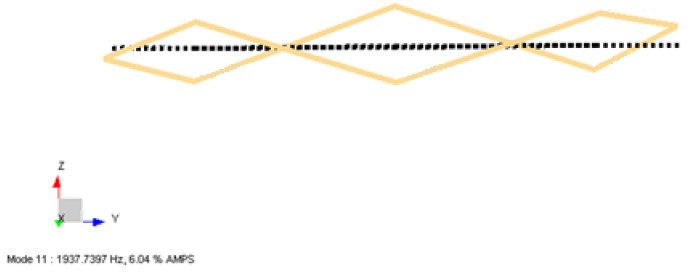
Third mode shape obtained from LMS Test Lab software for the Type-2 coil sample.

In Figure 14, Figure 15 and Figure 16, the orange lines represent the deformed shape, while the black dashed lines represent the undeformed shape of the Type-2 coil.

In Figure 14, the first bending mode of coil structure is obtained by processing both the –X and –Z excitation data merged. As expected, the first mode shape is a bending mode and it occurs at 614 Hz, as shown. The shape of the structure bends from two points, similar to the results in Figure 10.

In Figure 15, the first in-plane mode of coil structure is obtained by processing both the –X and –Z excitation data merged. As expected, the second mode shape occurs in the XY plane at 1062 Hz, as shown. The coil shows the same behavior compared with Figure 11, as expected.

In Figure 16, the second out-of-plane mode of coil structure is obtained by processing both the –X and –Z excitation data. The excitation test results are merged into one complete data set to calculate the global natural frequencies and mode shapes. The third mode shape occurs as the second bending mode at 1937 Hz, as shown. The shape of the structure bends from three points. These modes occur as twins close to each other due to the transition zone of the quadrupole winding method. However, it is very difficult to monitor the three-point out-of-plane bending with four moving accelerometers placed at 45-degree intervals. In this test, the merged excitation data does not give a natural frequency result close to the real situation. In this study, we are interested in the first two modes, eliminating their effects on sensor performance so the exact values are not a serious concern for the third mode.

The results found for each coil case are expected for a ring-shaped structure in which an out-of-plane mode occurs prior to the in-plane mode. Because of the elastic modulus of the glass fiber part being higher than the elastic modulus of adhesive part, the circumferential stiffness should be higher compared with the axial direction, and the collected results for both coil configurations in modal testing processes give a meaningful order for the mode shapes for a ring-shaped coil structure.

## 5. Finite Element Analysis Results

In order to obtain the orthotropic elastic properties of the FOG coil, an RVE approach is employed, as mentioned in Section 3. The global material properties calculated through the homogenization procedure are fed into the global FE model of the full FOG coil. The natural frequencies and mode shapes are calculated by means of modal analysis. After the comparison of the FOG coil modal analysis results with the modal test results, the homogenized orthotropic material properties of the FOG coil are verified.

### 5.1. RVE Analysis Results

In this subsection, the finite element analysis results of the RVE cell will be discussed. The components of tensor matrix C are used to find the homogeneous material properties.

**Figure 17:**
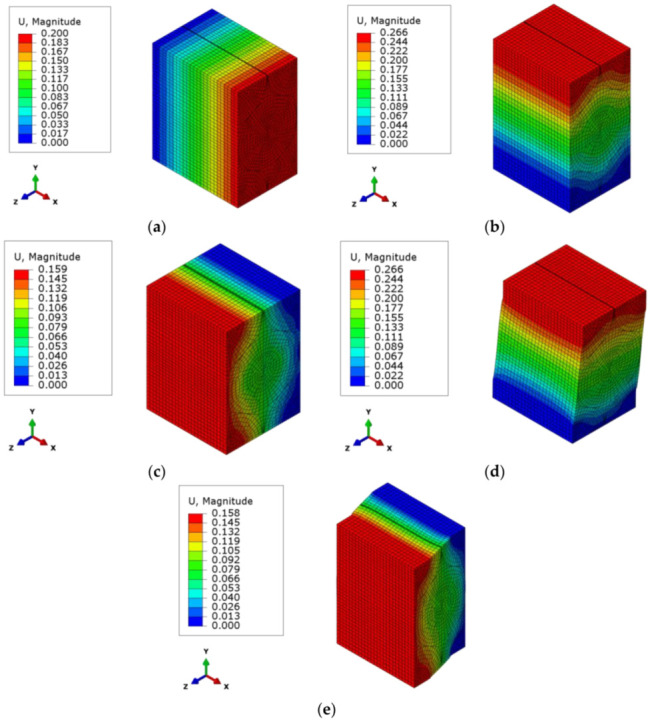
Displacement results (in mm) for the: (**a**) first column of tensor C; (**b**) second column of tensor C; (**c**) third column of tensor C; (**d**) fourth column of tensor C; and (**e**) sixth column of tensor C.

In the computation of the homogenized orthotropic material properties of the RVE, unit strains are applied in each direction by employing the boundary conditions explained in Section 3 (Figure 17). The calculated stress results in the elements of the RVE are integrated through the volume of the elements and divided by the total volume of the RVE cell. The compliance matrix of the RVE is obtained with this procedure. Using the elements of the compliance matrix, the equivalent homogenized orthotropic material properties are calculated in Equations (4)–(9). These results are tabulated in Table 4.

The homogenized orthotropic material properties given in Table 4 are used in the global coil FE model in cylindrical coordinates.

### 5.2. Modal Analysis Results of Global Coil FE Model

In this subsection, the global FE model results for each coil case are discussed. The calculated material properties from the RVE model are used as input parameters in the coil Type-1 global FE model. The global FE model contains the homogenized coil material and the geometrical properties for the coil.

**Figure 18:**
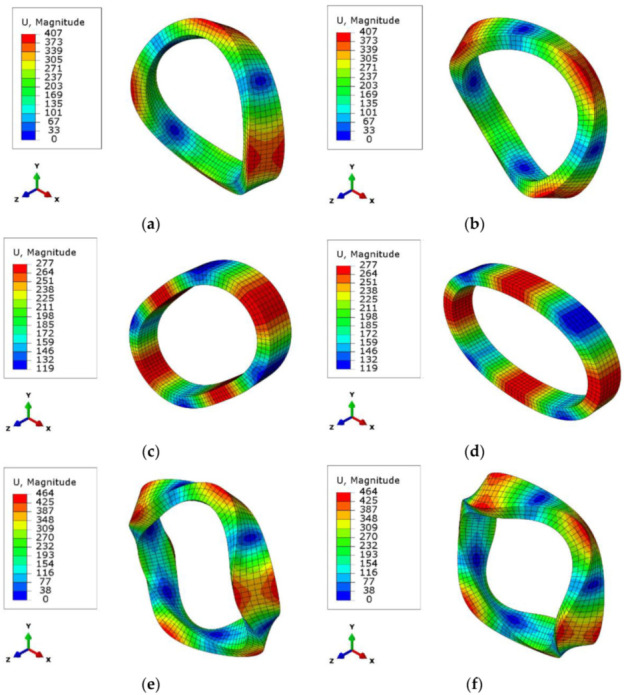
Mode shapes for the coil Type-1: (**a**,**b**) first mode shape; (**c**,**d**) second mode shape; (**e**,**f**) third mode shape.

The natural frequencies and mode shapes for the coil Type-1 from the global FE model are given below in Figure 18. The magnitude U is the displacement in microns.

The average results of the modal tests for the produced coil sets, which contain five coil samples, and the modal analysis results for the same coil in the finite element model are compared in Table 5. The percent error gives the percentage between the FE modal analysis and the modal test. The standard deviation values for the modal test natural frequencies of the five coils are 2.1%, 0.8%, and 2.9%, respectively, for mode 1, mode 2, and mode 3 for Type-1 coil fiber winding.

In Table 5, it can be seen that the first mode, which is the first out-of-plane movement, is approximated with an error below 1%. The second mode, which represents in-plane movements, is approximated at around a 10% error gap. In this global FE model, the jogging zone of the fibers are not modeled. The coil is assumed to be perfectly symmetric, which was not the real situation. The approximation of the first natural frequency is the most crucial one for the designer to shape the coil support for the coil geometry. This result says to the designer that the first mode shape dynamics in the coil are modeled with a correct approach compared with the real coil situation.

**Figure 19:**
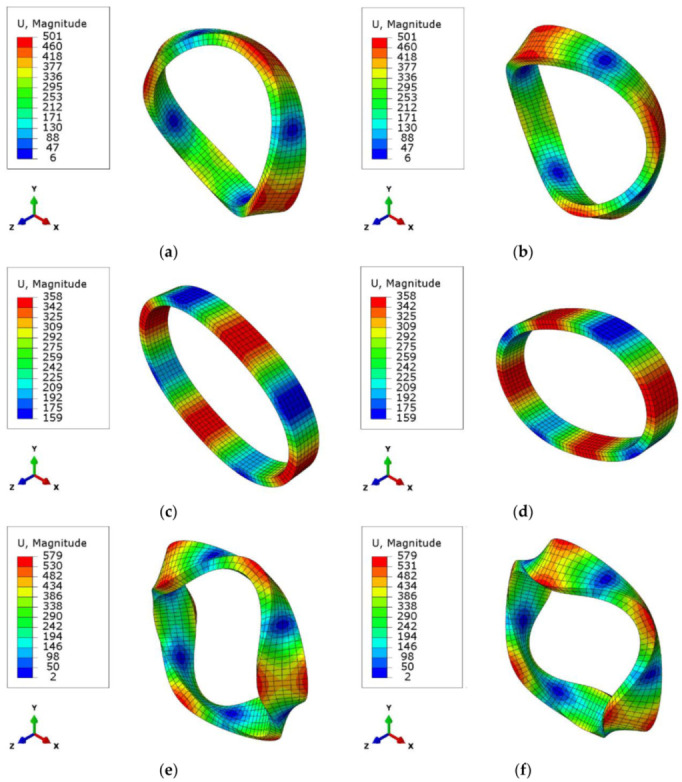
Mode shapes for the coil Type-2: (**a**,**b**) first mode shape; (**c**,**d**) second mode shape; (**e**,**f**) third mode shape.

The natural frequencies and mode shapes for a Type-2 coil are given below in Figure 19. The magnitude U is the displacement in microns.

The average results of the modal tests for the produced coil sets, which contain five coil samples, and the modal analysis results for the same coil in the finite element model are compared in Table 6. The standard deviation values for the modal test natural frequencies of five coils are 1.5%, 0.9%, and 2.7%, respectively, for mode 1, mode 2, and mode 3 for Type-1 coil fiber winding.

It can be seen from Table 6 that the first mode, which is an out-of-plane movement, is approximated with an error below 1%. The second mode, which represents in-plane movements, is approximated at around a 12% error gap.

For the modeling, the finite element (FE) method is used to construct a model based on the representative volume element in which the global parameters determined. The global model results of the FE model for Type-1 coil are then compared with the impact modal test results. Next, the Type-2 coil global FE model is constructed with the same homogenized RVE parameters and compared with the modal test results. The first natural frequencies of both structures are approximated below a 1% error compared with the produced coil configurations.

## 6. Conclusions

In this study, the dynamic responses of fiber-optic gyroscopes (FOGs) were studied through a multiscale modeling approach in comparison with experimental observations. Initially, a representative volume element was generated to obtain the macroscopic material parameters, including the influence of microstructure through a step-by-step approach with proper boundary conditions. The obtained parameters were used for the orthotropic material model, which was employed for the modal analysis of the FOG coil in ABAQUS software. The results were compared with the experimental findings and, after obtaining a satisfactory agreement, the approach was successfully applied for another FOG coil with a different fiber length, to increase the reliability of using such an approach for the analysis of FOGs in general. In this approach, a maximum error of below 10% between the results of FE modeling and modal tests was achieved. Moreover, the first natural frequencies of the structures were provided with an error less than 1%. Owing to this work, fiber-optic coils with different fiber lengths can be identified by means of the dynamic behavior for the estimation of coil support types and their effects on sensor performance. Pre-known coil mode shapes and their frequencies will provide information to the designer on whether they need to focus on a vibration rectification error (VRE) problem or not, even if the coil is not produced or tested at all. Therefore, the performance grade of the fiber-optic coil could be enhanced according to the needs of the platform.

In order to solve the complex orthotropic material behavior issue of the FOG coil, RVE-based multiscale analysis methodology was employed and the vibrational properties of multiscale FOG coils were obtained from both experimental modal analysis and finite element calculations. The novelty of the study is in the verification of the macroscopic material properties of the FOG coil obtained from the microstructure by means of vibration tests. Studies regarding the vibrational properties of composite materials are uncommon and, especially with regard to fiber-optic gyro sensors, an experimental modal analysis approach is rare in the literature. By using this procedure, more robust FOG designs can be achieved with regard to VRE problems.

The most important and valuable result of this study is that the dynamic behavior and support structure design requirements for FOG coils can be estimated and decisions can be made prior to the manufacturing and testing stages. Production and prototype testing costs may be reduced, or even totally avoided, with such global FE models for different geometric dimensions for the width or radius of coils that have different total fiber lengths. Hence, the designer can choose the available raw materials for coil supports and construct joint types between the support part and the coil part based on the geometry of the whole sensing assembly as a single complete sensing unit. The composite components of the coil and the coil support components are able to be combined in the same FE model for differing sensor grades.

The most complex and crucial part of the study is the coil due to its orthotropic nature. In this work, a comprehensive and experimentally verified model for a FOG coil has been established as a baseline study. The next stage of FOG sensor design is the simulation of the coil support together with the coil. As the coil support is mainly composed of isotropic materials, a straightforward simulation process that is less challenging can be used. As a future study, different types of coil support designs with different materials and geometries will be simulated with the FOG coil as a part of the sensor design.

## Figures and Tables

**Table 1:** Technical specifications of fiber.

Parameter	Fiber
Operating Wavelength (nm)	1550
Numerical Aperture	0.19–0.21
Mode Field Diameter (μm)	6.0–7.0 @1550 nm
Beat Length @633 nm (mm)	≤1.15
Proof Test (%)	1 (100 kpsi). 2 (200 kpsi) or greater upon request
Cladding Diameter (μm)	80 ± 1
Core Cladding Concentricity (μm)	≤1.0
Coating Diameter (μm)	155 ± 5
Coating Type	Dual Acrylate

**Table 2:** Dimensions for Type-1 quadrupole wound coils (Figure 1).

Inner Radius (mm)	Outer Radius (mm)	Thickness (mm)
32.3	38.35	11.9

**Table 3:** Dimensions for Type-2 quadrupole wound coils.

Inner Radius (mm)	Outer Radius (mm)	Thickness (mm)
32.3	36.05	11.9

**Table 4:** Global material properties obtained from the RVE model.

E_1_	E_2_	E_3_	*ν*_12_	*ν*_13_	ν_23_	G_12_	G_13_	G_23_
19,382	5852	5823	0.104	0.104	0.546	180	180	175

**Table 5:** Comparison of test and analysis results for the coil Type-1.

Test/Analysis	Mode 1	Mode 2	Mode 3
Modal Test	789.34 Hz	1389.48 Hz	2374.85 Hz
Modal Analysis	785.09 Hz	1532.70 Hz	2386.50 Hz
Percent Error	0.54%	10.31%	0.49%

**Table 6:** Comparison of test and analysis results for the coil Type-2.

Test/Analysis	Mode 1	Mode 2	Mode 3
Modal Test	618.17 Hz	1055.54 Hz	1814.25 Hz
Modal Analysis	612.94 Hz	1182.4 Hz	1987.55 Hz
Percent Error	0.85%	12.01%	9.55%

## Data Availability

The data presented in this study are available on request from the corresponding author. The data are not publicly available due to confidential information.
